# Deep Learning of Suboptimal Spirometry to Predict Respiratory Outcomes and Mortality

**DOI:** 10.21203/rs.3.rs-6296752/v1

**Published:** 2025-06-30

**Authors:** Davin Hill, Max Torop, Aria Masoomi, Peter J. Castaldi, Edwin K. Silverman, Sandeep Bodduluri, Surya P. Bhatt, Taedong Yun, Cory Y. McLean, Farhad Hormozdiari, Jennifer Dy, Michael H. Cho, Brian D. Hobbs

**Affiliations:** 1 Department of Electrical and Computer Engineering, Northeastern University, Boston, MA, USA.; 2 Channing Division of Network Medicine, Brigham and Women’s Hospital, Boston, MA, USA.; 3 Division of General Medicine and Primary Care, Brigham and Women’s Hospital, Boston, MA, USA.; 4 Harvard Medical School, Boston, MA, USA.; 5 Division of Pulmonary and Critical Care Medicine, Brigham and Women’s Hospital, Boston, MA, USA.; 6 Division of Pulmonary, Allergy and Critical Care Medicine, University of Alabama at Birmingham, Birmingham, AL, USA.; 7 Google Research, Cambridge, MA, USA.

## Abstract

**Importance:**

Obtaining spirometry requires repeated testing and using the maximal values based on quality control criteria. Whether the suboptimal efforts are useful for the prediction of respiratory outcomes is not clear.

**Objective:**

To determine whether a machine learning model could predict respiratory outcomes and mortality based on suboptimal spirometry.

**Design:**

Observational cohorts (UK Biobank and COPDGene).

**Setting:**

Multi-center; population, and disease-enriched.

**Participants:**

UK aged 40–69; US aged 45–80, >10 pack-years smoking, without respiratory diseases other than COPD or asthma.

**Exposures:**

Raw spirograms (volume-time).

**Main outcomes and measures:**

To create a combined representation of lung function we implemented a contrastive learning approach, **Spiro**gram-based **C**ontrastive **L**earning **F**ramework (Spiro-CLF), which utilized all recorded volume-time curves per participant and applied different transformations (e.g. flow-volume, flow-time). We defined “maximal” efforts as those passing quality control (QC) with the maximum FVC; all other efforts, including submaximal and QC-failing efforts, were defined as “suboptimal”. We trained the Spiro-CLF model using both maximal and suboptimal efforts from the UK Biobank. We tested the model in a held-out 20% testing UK Biobank subset and COPDGene, on 1) binary predictions of FEV_**1**_/FVC <0.7, and FEV_**1**_ Percent Predicted (FEV_**1**_PP) <80%, 2) Cox regression for all-cause mortality, and 3) prediction of respiratory phenotypes.

**Results:**

We trained Spiro-CLF on 940,705 volume-time curves from 352,684 UKB participants with 2–3 spirometry efforts per individual (66.7% with 3 efforts) and at least one QC-passing spirometry effort. Of all spirometry efforts, 61.6% were suboptimal (37.5% submaximal and 24.1% QC-failing). In the UK Biobank, Spiro-CLF using QC-failing and submaximal efforts predicted FEV_**1**_/FVC < 0.7 with an Area under the Receiver Operating Characteristics (AUROC) of 0.956, mortality with a concordance index of 0.647, and asthma with a 9–42% improvement versus baseline models. In COPDGene (n=10,110 participants), adding QC-passing, submaximal efforts did not improve the prediction of lung function or mortality; however, Spiro-CLF representations predicted asthma and respiratory phenotypes (joint test P ≤ **2** × **10**^−**3**^).

**Conclusions and Relevance:**

A machine-learning model can predict respiratory phenotypes using suboptimal spirometry; results from all spirometry efforts may contain valuable data. Additional studies are required to determine performance and utility in specific clinical scenarios.

## 1 Introduction

Spirometry is one of the most commonly used diagnostic tests in pulmonary medicine [1]. It is essential for diagnosing COPD and plays a critical role in assessing asthma, interstitial lung disease, neuromuscular conditions, preoperative pulmonary risk, and monitoring for drug toxicity and environmental exposures [2]. Annual spirometry is recommended for asthma and COPD [3], which together affect over 200 million people worldwide [4, 5].

Spirometry is performed by an individual taking a maximum inhalation followed by a maximum forced exhalation into a spirometry device, producing a graphical volume-time curve known as a spirogram. Spirograms are used to calculate key spirometry measurements, including forced expiratory volume in 1 second (FEV_1_), forced vital capacity (FVC), the FEV_1_/FVC ratio, and forced mid-expiratory flow (FEF) [6].

However, spirometry measurements are often noisy, with repeated efforts yielding variable measurements [7]. Unlike most biomedical measurements, spirometry depends heavily on effort and requires repeated attempts to ensure reliability. Clinical guidelines mandate at least two high-quality, reproducible efforts with minimal variability in measured volumes, though three or more attempts are commonly performed. [6, 8] The “best” spirometry measurements are selected as the highest values recorded across all high-quality, reproducible efforts. Spirometry quality control (QC) guidelines include factors such as minimal back-extrapolated volume (i.e., assuring the forced exhalation effort is maximal from the very start of the exhalation maneuver), sufficient amount of time performing the forced exhalation, and a sufficient flow plateau at the end of the forced exhalation effort. Any spirometry efforts that are not reproducible or do not meet QC guidelines are discarded, since traditional spirometry measurements are only extracted from the QC-passing, maximal efforts.

We hypothesize that the suboptimal (i.e. submaximal and QC-failing) spirometry efforts carry valuable information about lung function and overall health outcomes, which can be effectively captured using deep learning methods trained on the entirety of the spirometry curves. While the suboptimal efforts may be less informative compared with the maximal effort, it may remain relevant for understanding overall lung function. For example, the suboptimal efforts contain information related to the variance and reproducibility of the reported spirometry measures; the existence of failed efforts may be correlated to underlying lung function and pulmonary disease. Additionally, the combined information from the suboptimal efforts may help reconstruct the information from the maximal effort, potentially reducing the number of efforts required during a clinical visit.

Therefore, the objective of this study was to evaluate whether information derived from suboptimal spirometry efforts could provide comparable or even superior relevance for lung function and mortality prediction. To do this, we developed a deep learning method, the **Spiro**gram-based **C**ontrastive **L**earning **F**ramework (Spiro-CLF), to learn nonlinear vector representations of lung function from the entirety of an individual’s spirometry efforts, including suboptimal efforts. Spiro-CLF does not require any manual feature engineering or annotation of the spirograms, and is trained using a self-supervised, contrastive learning approach [9].

We trained the Spiro-CLF model on raw, pre-bronchodilator spirograms from the UK Biobank study (UKB) and assessed the quality of the learned vector representations on a held-out UK Biobank test dataset across three prediction tasks: (1) prevalence of lung function impairment, (2) mortality risk prediction, and (3) prediction of respiratory phenotypes. In each task, the vector representations of lung function served as substitutes for traditional spirometry measures. We additionally applied the Spiro-CLF model on the Genetic Epidemiology of COPD Study (COPDGene) to further validate the quality of the extracted representations on a separate cohort.

Our results showed that including the suboptimal spirometry efforts led to improved prediction of lung function impairment, mortality prediction, and phenotype prediction compared to both utilizing only maximal efforts and classic summary measures of lung function from spirometry. The Spiro-CLF model was able to be applied directly to the COPDGene dataset without any additional training, demonstrating the generalizability of the model to other datasets. These results indicate that Spiro-CLF is able to derive vector representations of lung function from suboptimal spirometry that are more predictive of clinical outcomes than traditional spirometry measures.

## 2 Methods

### 2.1 UKB Spirogram Preprocessing

UKB participants were recruited across 21 assessment centers in the United Kingdom for the UK Biobank database. Additional details regarding the UK Biobank study were previously published in [10]. A summary of participant characteristics is listed in Table 1. We extracted raw, pre-bronchodilator spirograms from UK Biobank field 3066. We utilized the spirograms from the initial visit for each participant, thus excluding multiple visits from the same participant. Spirograms were recorded as total exhaled lung volumes (mL) at 10 ms intervals over a median of 7.9 seconds. Each participant had between one and three spirometry efforts.

**Fig. 1:**
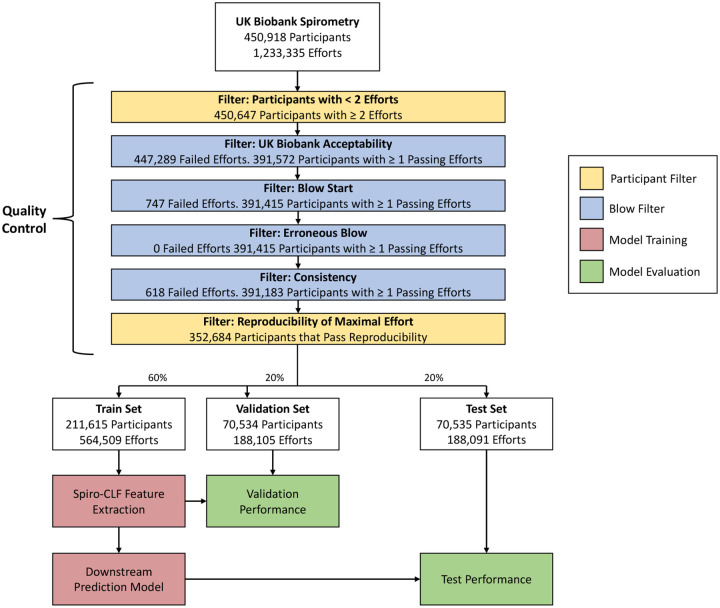
Overview of the Quality Control (QC), Spiro-CLF training, and downstream prediction process. The QC process contains participant filters, which contains participant-level criteria, and blow filters, which contain effort-level criteria. In each blow filter, spirometry efforts are tested against the specified criteria and labeled as either passing or failing the given filter. Participants with less than one QC-passing efforts or failing to produce a reproducible effort are removed from the dataset. QC criteria details are provided in Table 3.

**Fig. 2:**
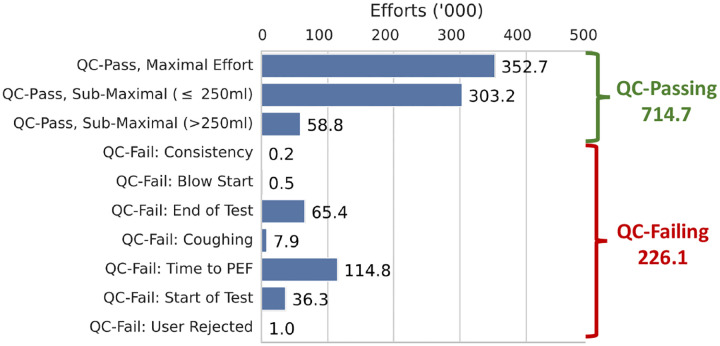
Number of UKB efforts after preprocessing, listed by effort type. *QC-Pass, Submaximal* ≤ *250mL* denotes efforts that pass QC with FVC within 250mL of the maximal FVC.

The following quality control filtering and preprocessing steps were applied to the spirogram samples. We used the spirometry quality control as previously outlined by Shrine et al. [11], which keeps key components of the ATS and ERS guidelines with modifications to retain additional samples. Specifically, a more liberal threshold for repeatability was used, which is explained in further detail in Shrine et al. [11]. We considered failing QC according to the steps outlined in Table 3. We considered efforts passing these QC criteria, but not corresponding to the maximal effort, “submaximal”. For model validation purposes, we required all participants to have at least one QC-passing, reproducible effort. Effort reproducibility was defined as the presence of a second effort that achieved an FVC within 250mL of the maximal FVC. Spirometry efforts were flagged as passing or failing each step of the QC process (Fig. 1). After each step, participants with less than one passing effort were removed from the dataset. After removing the participants with less than one QC-passing effort or fail the reproducibility criteria, the entirety of the efforts from remaining participants are included in the dataset. The distribution of remaining efforts is shown in Fig. 2.

After the QC process, the raw spirograms was downsampled from 10ms to 60ms intervals and the exhalation duration was limited to 30s to improve model efficiency. Efforts were truncated after reaching FVC; we replaced the spirometry volume values after achieving FVC to be the total spirogram FVC.

### 2.2 COPDGene Spirogram Preprocessing

The Genetic Epidemiology of COPD Study (COPDGene) is an observational study which contains detailed genetic, phenotyping, and spirogram data for over 10,000 participants [12]. A summary of participant characteristics is listed in Table 2. We utilized the spirograms recorded from each patient’s initial visit. The raw spirograms were recorded as total exhaled volume (mL) in 60ms intervals. We follow the UKB preprocessing procedure by limiting spirograms to 30s, and replacing spirometry volume values after achieving FVC to be the total spirogram FVC. In contrast to UKB, the spirograms in COPDGene were already processed with automated and human annotation to remove QC-failing efforts. Therefore, each participant in COPDGene had between 2–3 spirometry efforts (97.0% with 3 efforts), with all efforts (n=30,012) passing QC.

### 2.3 Overview of Spiro-CLF

**Fig. 3:**
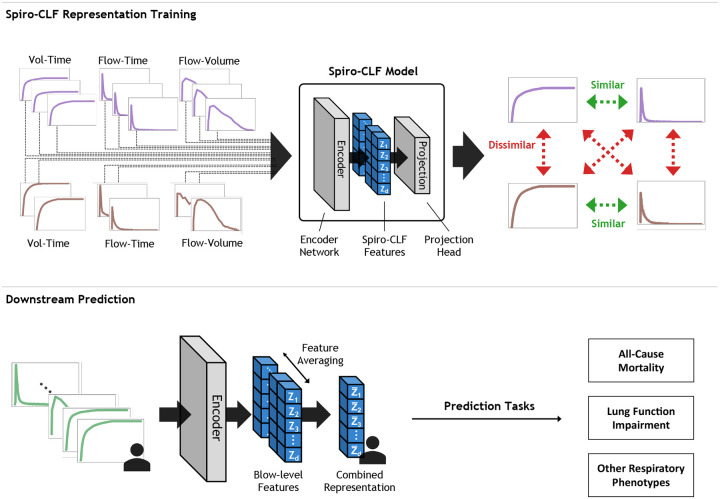
**Top:** Schematic of the Spiro-CLF training process. We randomly applied flow-time, flow-volume, and identity (volume-time) transformations to each spirometry effort within the training batch. The Spiro-CLF model is trained with contrastive loss to predict high pairwise similarity between efforts from the same individual and low pairwise similarity between efforts from different individuals. **Bottom:** Once the Spiro-CLF model is trained, we applied the encoder network to the entirety of an individual’s efforts, including transformation, to generate a single feature representation for each individual. This representation can then be used in a variety of downstream predictive tasks.

The Spiro-CLF framework consists of two stages (Fig. 3). First, the Spiro-CLF model was trained using a self-supervised, contrastive learning approach^1^ [9]. The Spiro-CLF model consists of a Convolutional Neural Network (CNN) [13] encoder, which maps the spirogram inputs to a vector representation, and a Multi-Layer Perceptron (MLP) projection head, which maps the vector representation to a prediction space. During training, the volume-time spirograms were augmented with random transformations, including flow-time and flow-volume transformations, to increase the diversity of the training data. A binary encoding, indicating transformation type, was appended to the end of each spirogram sample. The Spiro-CLF model was then trained to cluster spirograms from the same participant together in feature space, while separating spirograms from different participants, using the NT-XENT contrastive loss function [9]. This training procedure encouraged the Spiro-CLF model to identify the unique characteristics of each participant’s lung function contained within the entirety of the patient’s spirometry efforts. Spiro-CLF was trained on a randomly selected 60% subset of the UKB dataset. Model hyperparameters were selected using a separate 20% validation set.

In the second stage, the trained encoder was used to extract the learned vector representations for each participant. The encoder network processed the entirety of an individual’s spirometry efforts, generating separate representations for each spirogram and transformation. These representations were then averaged to produce a single vector representation of lung function for each participant, which we refer to as the Spiro-CLF representation

To evaluate the performance of Spiro-CLF, we utilized three separate test sets that were excluded from the training process: 1) a randomly selected 20% subset of the UK Biobank dataset, 2) samples from two held-out UK Biobank assessment centers, and 3) the COPDGene cohort. The latter two groups served as external replication datasets to validate the model’s generalizability. During Spiro-CLF validation, we generated vector representations for each participant in both training and test sets. For each prediction task, we then trained linear prediction models on the vector representations from the training set, then evaluated model performance on the respective test sets.

Further method details, background on contrastive learning, and additional results can be found in the supplement (App. D, E, F).

## 3 Results

### 3.1 Lung Function Impairment Prediction with Spiro-CLF Representations

**Fig. 4:**
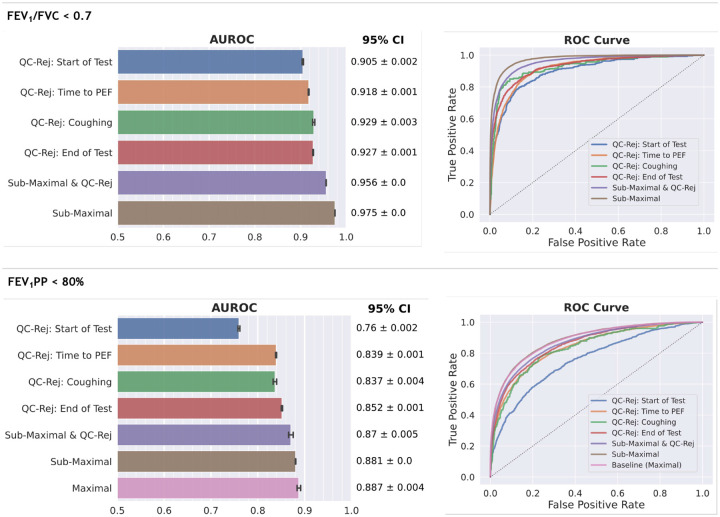
Results of the lung function impairment prediction tasks: FEV_1_/FVC< 0.7 (Top), and FEV1PP< 80% (Bottom). The error bars represent 95% bootstrap confidence intervals (50 iterations).

Lung function impairment is often defined using FEV_1_/FVC and FEV_1_PP metrics, derived from the maximal, QC-passing effort [1]. We evaluated the ability of the Spiro-CLF vector representations to predict lung function impairment when restricted to using submaximal and QC-failing efforts. This simulates a scenario where only suboptimal spirometry data is available for a given individual. We included two separate prediction tasks: A) FEV_1_/FVC< 0.7 and B) FEV_1_PP< 80%. Spiro-CLF was calculated using GLI-2012 reference values [14] with covariates sex, height, age, and self-reported ethnicity. Covariates were excluded from the prediction model in order to directly evaluate the Spiro-CLF representations. Results are shown in Figure 4. We omitted COPDGene results from this section, since COPDGene does not include QC-failing spirograms.

We observed that the predictive performance of the Spiro-CLF representations on QC-failing and submaximal efforts was significantly recovered as compared to the baseline of using the maximal effort on both the FEV_1_/FVC and FEV_1_PP prediction tasks. In the FEV_1_/FVC task, the Spiro-CLF representations for submaximal and QC-failing efforts achieved 0.956 AUROC. Exclusively using submaximal efforts yielded AUROC of 0.975. Similarly, in the FEV_1_PP prediction task we compared the performance of the submaximal and QC-failing features to a baseline performance of using only features from maximal efforts. Note that the FEV_1_PP “maximal” baseline did not achieve 100% AUROC performance due to the omitted covariate information from the prediction model. Using the Spiro-CLF features achieved 0.870 (submaximal and QC-failing efforts) and 0.881 (submaximal efforts) AUROC compared to the baseline of 0.887 AUROC.

We further assessed the performance of the Spiro-CLF model using only efforts that failed QC. When restricted to efforts failing due to excessive time to peak expiratory flow (PEF), the model achieved an AUROC of 0.918 for task A and 0.839 for task B.

### 3.2 All-Cause Mortality Prediction with Spiro-CLF Features

We used a mortality prediction task to validate that the Spiro-CLF representation contains additional relevant information for disease progression and health outcomes. We trained Cox regression models^2^ on the Spiro-CLF representations for the UKB and COPDGene training partitions. The resulting model fit was evaluated on the respective test partitions using the concordance index (c-index). For comparison, we trained additional Cox models on alternative spirometry metrics. No additional covariates were included in the model training in order to compare the predictive power of each metric.

**Fig. 5:**
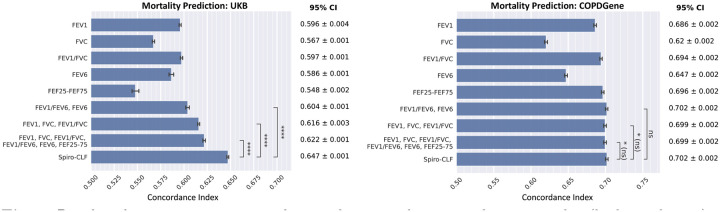
Results of a time-to-event mortality prediction task measured using c-index (higher is better). We trained the Cox regression model using different lung function representations to compare their predictive power with respect to mortality prediction. The error bars represent 95% bootstrap confidence intervals (50 iterations). **** indicates significance at the *α* = 10^−3^ significance level, with Holm-Bonferroni adjustment.

Results are shown in Figure 5. In the UKB dataset, Spiro-CLF Cox model achieved a c-index of 0.647, which surpassed the performance of the second-highest performing single metric, FEV_1_/FVC, with c-index 0.597 (P ≤ 1.4 × 10^−37^). By combining metrics FEV_1_, FVC, and FEV_1_/FVC, we increased the c-index to 0.616. Including all competing metrics in the Cox model further improved c-index to 0.622, which was still exceeded in performance by the Spiro-CLF features (P ≤ 8.3×10^−23^). We also evaluated Spiro-CLF performance using two UKB assessment centers (Bristol and Leeds) as the testing set. Results were consistent with the previous findings (App. F.6).

Spiro-CLF Cox model acheived a 0.702 c-index on the COPDGene dataset. In contrast to the UKB results, the Spiro-CLF Cox model did not show statistically significant improvement in c-index performance over the Cox models with combined spirometry metrics. The Spiro-CLF Cox model exhibited a mild performance increase over the best-performing single-metric Cox models.

### 3.3 Phenotype Prediction

We used a Phenotype prediction task to evaluate the predictive power of the Spiro-CLF representations with respect to various phenotypes related to lung function. We trained generalized linear models (GLM) on 13 different phenotypes (App. D.5.3) with 4 different sets of base predictors:

1. **Intercept-Only.** No predictors were included. The linear model was fit using only the intercept term.
2. **Covariates.** We included relevant covariates for each phenotype, as specified in Table 4. Covariates were selected based on prior work in COPDGene phenotype association [15].
3. **Spirogram Metrics.** We included traditional spirometry metrics FEV_1_, FVC, and FEV_1_/FVC, taken from each participant’s maximal QC-passing effort.
4. **Covariates & Spirogram Metrics.** All previously specified covariates and spirogram metrics are included.

**Fig. 6:**
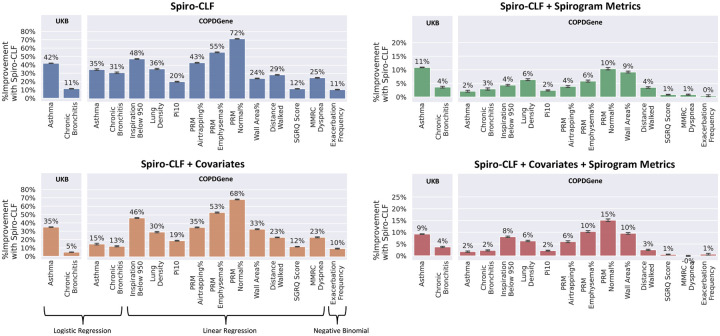
Improvement in model fit when combining Spiro-CLF vector representations with different sets of base predictors (Covariates, Spirogram Metrics, and both Covariates & Spirogram Metrics) to predict respiratory phenotypes. Model fit is evaluated using AUROC for Logistic Regression models and MSE for all other models. Error bars represent 95% bootstrap confidence intervals (50 iterations).

For each combination of phenotype and set of predictors, we trained two GLMs including and excluding Spiro-CLF features (m = 120 models in total). Model fit was evaluated using AUROC for binary phenotypes (Asthma, Chronic Bronichitis) and Mean Squared Error (MSE) for all other phenotypes. We then compared the improvement in model fit when including Spiro-CLF representations (Figure 6).

We observed that, using Spiro-CLF alone, we could improve prediction of all measured phenotypes by at least 11%. SpiroCLF also improved prediction over standard SM alone, and spirometry features and covariates. In COPDGene, we found a 15% improvement for prediction of normal lung on CT scan. For prediction of asthma, inclusion of spirometry led to minimal improvement in COPDGene (2% with covariates), but a 9% improvement in asthma in UKBB. Additionally, the Spiro-CLF features were jointly significant for all sets of predictors (P ≤ 2 × 10^−3^, App. F.5)

## 4 Discussion

The clinical test of spirometry involves repeating efforts and choosing specific features of the best results for clinical reporting. In this work, we show that a deep learning model, trained on repeated measures of the raw data, can improve prediction of relevant respiratory outcomes.

In the population-based UK Biobank sample, we demonstrate significant prediction of lung function impairment, mortality, and respiratory phenotypes using QC-failed efforts. In COPDGene, which only had QC-passed, reviewed, submaximal efforts, we demonstrate prediction of respiratory phenotypes. Our results suggest that the benefit of Spiro-CLF will depend on whether suboptimal efforts are available.

Our work complements other efforts in applying deep learning to raw spirometry curves for different tasks. Supervised deep learning models have been developed for applications such as predicting Chronic Obstructive Pulmonary Disease (COPD) risk [16, 17], COPD subtyping [18], prediction of upper airway obstruction [19], or acceptability criteria [20–22]. In contrast, our approach leverages unsupervised methods to learn a deep representation of lung function. Spiro-CLF uses a contrastive learning framework to generate a single representation using the entirety of an individual’s blows. This differs from other approaches [23] using Variational Autoencoders [24], which only use a single effort per individual.

Spiro-CLF features are a representation of lung function that are trained to be robust to noise inherent in the spirometry testing process. Therefore the Spiro-CLF features can be calculated for any effort from a given individual, including QC-failing and submaximal efforts. Our model is able to maintain high prediction of lung function impairment using QC-failing and submaximal efforts, and improve prediction of mortality when including the entirety of an individual’s efforts. In addition, the Spiro-CLF representation is generated from an unsupervised model which can be easily transferred across datasets. The resulting vector representation can directly replace traditional spirometry measures in any prediction task. Our findings suggest that Spiro-CLF or similar machine learning models could augment current clinical spirometry testing by providing a more accurate prediction of airflow limitation, lung function, and other outcomes, even in the absence of QC-passing, reproducible spirometry. Specifically, our model or similar models may be able to provide a prediction of reduced FEV_1_/FVC ratio or reduced FEV_1_ when QC-passing results are not available; in addition, our model can provide improved predictions of respiratory phenotypes, such as asthma or emphysema, beyond standard measures of lung function.

There are a number of possible causes for the improved predictive performance gained from using the Spiro-CLF representation. First, the Spiro-CLF encoding incorporates information from suboptimal spirograms; in particular, the QC-failing efforts may indicate underlying lung function impairment. For instance, elderly patients, or those with severe lung disease, may have difficulty performing a traditional FVC measurement appropriately and thus their spirometry efforts may be more likely to fail QC [25–28]. In addition, QC-passing, submaximal efforts contribute information about the variance relative to the maximal effort, offering insights not captured by maximal efforts alone.

The neural network parametrization of the Spiro-CLF model further improves predictive performance by encoding nonlinear effects from the raw spirograms. Previous works have shown that machine learning methods can be applied to raw spirograms to improve various prediction tasks related to COPD [16], COPD subtypes [18], and genetic association [17, 23].

Another possible factor in improved performance is the increase in training samples from utilizing the entirety of an individual’s efforts and additionally applying data transformations to obtain flow-time, flow-volume, and volume-time views of each effort. The combined efforts and transformations effectively increase the number of training samples per individual, up to 9x more when compared with using the maximal effort and a single blow representation. Deep learning models are known to require a significant number of data samples during training [29] and increasing the number of samples and the variability in data views offered by volume-time transformations to flow-volume and flow-time enables the use of more complex models and reduces the likelihood of over fitting the training data. We were additionally able to show that the trained Spiro-CLF model was able to be applied to smaller datasets, such as COPDGene, that may not necessarily have a sufficient dataset size to train a deep learning model from scratch with the same level of performance.

While we obtained QC-failing efforts from multiple testing centers in UK Biobank, we do not know whether these results are generalizable to QC-failing efforts using other spirometers in other scenarios of clinical care. In order to compare our models to “ground truth”, we excluded participants that were unable to perform at least one QC-passing spirometry effort. The UKB study was predominantly of self-identified white race, European genetic ancestry, and - as a volunteer cohort - has a “healthy volunteer” selection bias, while COPDGene is comprised of smokers with at least 10-pack-years. While we provide some assessment of features, showing that the more important features of our model are from the beginning of the blow, and we additionally predict normal (non-emphysematous or gas trapping lung), the specific physiologic parameters which Spiro-CLF is using for prediction are not known. Our results also do not suggest that existing lung function interpretation guidelines should change; in fact, the FEV_1_, FVC, and FEV_1_/FVC ratio as summative measures have advantages of simplicity and interpretability [30]. We did not evaluate performance of our model in other scenarios. Additional training may be needed when applying the Spiro-CLF model to settings including clinical spirometry. During training, additional validation may also be needed to ensure that model parameter and architecture choices are optimal with respect to the new dataset.

In conclusion, we developed a deep learning model leveraging repeated raw spirograms to predict lung impairment, mortality, and respiratory phenotypes. Our work underscores the richness of data embedded in spirometry beyond traditional metrics and highlights the potential of machine learning to maximize the use of this data.

## Supplementary Material

1

## Figures and Tables

**Table 1:** Characteristics of participants in the UK Biobank study.

Characteristic	Training Set	Validation Set	Test Set
Participants, n	211,603	70,534	70,535
Spirometry Efforts, n	564,509	188,105	188,091
Participants with 3 Efforts, n (%)	141,279 (66.8)	47,037 (66.7)	47,021 (66.7)
Age, yr, mean (SD)	56.3 (8.1)	56.3 (8.1)	56.3 (8.1)
Sex, F, n (%)	118,064 (55.8)	39,528 (56.0)	39,454 (55.9)
Ethnicity, Self-Reported			
Asian or Asian British, n (%)	3,564 (1.7)	1,266 (1.8)	1,276 (1.8)
Black or Black British, n (%)	2,752 (1.3)	939 (1.3)	938 (1.3)
Chinese, n (%)	606 (0.3)	209 (0.3)	207 (0.3)
Other, n (%)	2928 (1.4)	931 (1.3)	930 (1.3)
White, n (%)	201,074 (95.3)	66,968 (95.2)	66,963 (95.2)
Ever Smoke, n (%)	96,277 (45.5)	32,039 (45.4)	32,171 (45.6)
FEV1 / FVC, mean (SD)	0.74 (0.06)	0.74 (0.06)	0.74 (0.06)
Death Events, n (%)	7,376 (3.5)	2,536 (3.6)	2,478 (3.5)
Time to Event, yr, mean (SD)	11.2 (1.5)	11.2 (1.5)	11.2 (1.5)

**Table 2:** Characteristics of participants in the COPDGene study.

Characteristic	Training Set	Validation Set	Test Set
Participants, n	6,066	2,022	2,022
Spirometry Efforts, n	18,004	6,000	6,008
Maximal Efforts, n (%)	6,066 (33.7)	2,022 (33.7)	2,022 (33.7)
Submaximal Efforts, n (%)	11,938 (66.3)	3,978 (66.3)	3,986 (66.3)
Participants with 3 Efforts, n (%)	5,880 (96.9)	1,959 (96.9)	1,965 (97.2)
Age, yr, mean (SD)	59.5 (9.0)	59.3 (8.9)	59.7 (9.3)
Sex, F, n (%)	2,807 (46.3)	980 (48.4)	945 (46.7)
Ethnicity, Self-Reported			
African American, n (%)	1,979 (32.6)	692 (34.2)	694 (34.3)
Non-Hispanic White, n (%)	4,087 (67.4)	1,330 (65.8)	1,328 (65.7)
FEV1 / FVC, mean (SD)	0.65 (0.16)	0.66 (0.15)	0.65 (0.16)
Death Events, n (%)	1,596 (26.3)	510 (25.2)	544 (26.9)
Time to Event, yr, mean (SD)	8.7 (4.4)	8.7 (4.4)	8.8 (4.3)

## Key Points

### Question:

Can suboptimal spirometry data predict respiratory outcomes?

### Findings:

We analyzed raw spirogram curves, including efforts that failed quality control from the UK Biobank, as well as submaximal efforts from both the UK Biobank and COPDGene cohorts, using the Spirogram-based Contrastive Learning Framework (Spiro-CLF). Spiro-CLF predicted asthma and other respiratory phenotypes beyond standard lung function measures, and, in UK Biobank, predicted lung function impairment and all-cause mortality.

### Meaning:

This study demonstrates that suboptimal spirometry efforts can significantly contribute to predicting respiratory outcomes.

## Footnotes

1 We provide a review of contrastive learning methods in App. E.1.

2 We provide a review on Cox regression models and concordance index in App. E
