# Supplementary material for: Deep Learning of Suboptimal Spirometry to Predict Respiratory Outcomes and Mortality
Source: Res Sq. 2025 Jun 30:rs.3.rs-6296752. Preprint. [Version 1] doi: 10.21203/rs.3.rs-6296752/v1 (PMC12236919; doi:10.21203/rs.3.rs-6296752/v1)
Supplement: 1 [file NIHPPrs6296752V1-supplement-1.pdf]

APPENDIX

D Method Details

D.1 UKB Spirogram QC Criteria

In Table 3 we detail the QC criteria used for UKB spirograms during preprocessing.

D.2 Representation Learning with Spiro-CLF

The aim of our study was to uncover the complex, non-linear relationships between suboptimal spirometry data and the derived spirometry metrics, i.e. FEV_1 and FVC, for UK Biobank participants. The raw spirometry efforts are often "noisy", in that the observed efforts can be characterized as noisy signals from some underlying lung function. We therefore introduced a contrastive learning approach,

QC Filter	Description
Participants with <2 Efforts	Removes participants with less than two recorded efforts.
UK Biobank Acceptability	Fails efforts that are flagged in UK Biobank Field 3061 as START_OF_TEST (0x01), TIME_TO_PEF (0x02), END_OF_TEST (0x04), COUGHING (0x08), USER_REJECTED (0x40).
Blow Start	Fails efforts with excessive back extrapolated volume. Back extrapolated volume must be less than 5% of FVC or 150mL of total volume, whichever is greater.
Erroneous Blow	Fails efforts with invalid values for FEF25, FEF75, FEF25-75, PEF volume, back extrapolated volume, or FET volume.
Consistency	Fails efforts with inconsistency between derived FEV1, FVC values and UK Biobank recorded values. Derived FEV1 (resp. FVC) must be within 5% of UK Biobank field 3063 (resp. 3062)
Reproducibility	Removes participants unable to reproduce the maximal effort. Participants must have at least one effort with FVC less than the maximal FVC and greater than the maximal FVC less 0.25L.

Table 3: QC filter description for preprocessing participants and spirometry efforts from the UK Biobank.

Spirogram-based **C**ontrastive **L**earning **F**ramework (Spiro-CLF) to identify a feature representation that is invariant to noise in each individual effort, thereby capturing the underlying factors that contribute to lung function.

Contrastive learning is a self-supervised approach that has recently shown to be effective in improving image and language processing prediction tasks [31, 32]. It typically involves two steps: 1) an initial prediction task, often called the *pretext* task, that learns a feature representation of the data, and 2) the *downstream* task, which uses the learned representation on the ultimate prediction goal. The pretext task is self-supervised in that the model is discriminative, i.e. it is trained to predict a given label, however the pretext labels are derived entirely from the dataset itself.

Our work is most closely related to Chen et al. [9], which was introduced in the context of image classification, and uses different correlated transformations of a given image as input to a deep learning model. Spiro-CLF defines each data sample as an individual participant, and each spirometry effort as different perspectives of that participant's lung function. We additionally applied transformations (e.g. flow-volume, flow-time) to the spirometry efforts to further improve model performance; we define these transformations in section D.4.

An overview of the Spiro-CLF representation learning process is shown in Figure 3. During training, Spiro-CLF sampled randomized batches of spirometry efforts. Each batch contained pairs of spirometry efforts produced from the same individual. Each effort within the batch had one positive example belonging to the same individual, and $B-2$ negative examples, where B is the batch size. The model predicted whether any two given efforts within the batch were produced by the same individual or different individuals, represented by a predicted similarity score for each pair of efforts. The contrastive loss encouraged the model to predict high similarity for efforts produced from the same individual, and low similarity for efforts from disparate individuals.

We parameterized the Spiro-CLF model using a Convolution Neural Network (CNN) architecture, which captures the temporal relationship between each time step in each sample. CNN models have been previously shown to be highly effective on time-series prediction tasks [33, 34]. The convolution layers within CNNs encode the assumption that the relationship between values at adjacent time steps are more relevant than values between non-adjacent time steps. The model architecture and training process is defined in more detail in section D.3.

Once the model was trained, we used the output of an intermediate layer as the learned representation of a participant's overall lung function. We incorporated a feature averaging step [35] when generating Spiro-CLF features to further encourage invariance of the learned representations. During this step, we sampled the entirety of transformed efforts from each individual and averaged the respective features. The final representation can then be used in downstream prediction tasks

All model training and experiments were performed on a computing cluster using AMD EPYC 7302 16-Core processors and NVIDIA A100 GPUs. All source code is available at https://github.com/davinhill/Spiro-CLF.

D.3 Spiro-CLF Model Architecture

We parametrized the Spiro-CLF model with a deep neural network. Model architecture and hyperparameters were selected using a randomized grid search procedure, optimized with respect to minimum loss on the validation set. The model consisted of six convolution blocks; each block contained a 1-dimensional convolution layer with weight normalization, ReLU activation function, and skip-connection. Each convolution had a fixed kernel size of 40 with padding to avoid downsampling. After the convolution blocks, two fully-connected layers were applied to downsample to the 100-dimensional feature space. We trained an additional projection head parametrized by a multi-layer perceptron (MLP) with one hidden layer, ReLU activation, and batch normalization. The projection head maps the feature space to a 128-dimensional output to calculate contrastive loss. The detailed model architecture is shown in Figure 7.

We trained the model for 600 epochs to minimize the contrastive loss of the projection head output. We utilised the Normalized Temperature-scaled Cross Entropy (NT-XENT) loss function [9] to calculate contrastive loss. During training, the contrastive loss was calculated using batches of 1024 spirograms, which included two transformed spirograms from each of 512 randomly selected participants. The available transformations are discussed in section D.4. The model was trained using the Adam optimizer [36] with an initial learning rate of 10^{-2} and a scale factor of 0.1 on validation loss plateau.

After training, the projection head was discarded, and the model was applied to the spirometry samples in the test set to calculate the Spiro-CLF features.

D.4 Sampling of Spirometry Efforts and Transformations

During Spiro-CLF model training, we randomly selected two efforts from each participant in a given batch of participants. The two efforts were sampled uniformly from the participant's collective spirometry efforts. If a participant had exactly two efforts, then both efforts were used in the training batch. We then applied a random data transformation to each effort.

During the data transformation stage, we applied either a flow-time transformation, flow-volume transformation, or identity (volume-time) transformation, selected with uniform probability. These transformations are detailed below. After each transformation, we appended a 3-dimensional binary vector to each blow sample indicating which transformation was applied. Therefore, in each training batch, each participant had two separate spirometry efforts, which were represented by either volume-time, flow-time, or flow-volume curves.

- **Volume-Time.** No additional transformation was needed to obtain volume-time representations. The original spirograms were saved in 10ms (UKB) or 60ms (COPDGene) intervals intervals; we downsampled all volume-time curves to 60ms time intervals to allow model compatibility between both UKB and COPDGene datasets.
- **Flow-Time.** We applied a flow-time transformation to the original flow-time data by calculating the change in expiratory volume, using a 120ms moving average. The resulting flow-time curves were downsampled to 60ms time intervals.
- **Flow-Volume.** We applied a flow-volume transformation to the original flow-time data to calculate spirogram flow at 50ms volume intervals. Due to the 10ms (60ms for COPDGene) sampling interval in the original volume-time data, the exact flow at each 50ml volume interval was not always available; we used linear interpolation to estimate the flow at those missing intervals.

```
CNN(
  (conv_encode): Sequential(
    (0): ConvBlock(
      (conv): Conv1d(1, 200, kernel_size=(40,), stride=(1,), padding=(39,))
      (dropout): Dropout(p=0, inplace=False)
      (downsample): Linear(in_features=1, out_features=200, bias=True)
      (bn): BatchNorm1d(200, eps=1e-05, momentum=0.1, affine=True,
          track_running_stats=True)
    )
    (1): ConvBlock(
      (conv): Conv1d(200, 200, kernel_size=(40,), stride=(1,), padding=(39,))
      (dropout): Dropout(p=0, inplace=False)
      (downsample): Linear(in_features=200, out_features=200, bias=True)
      (bn): BatchNorm1d(200, eps=1e-05, momentum=0.1, affine=True,
          track_running_stats=True)
    )
    (2): ConvBlock(
      (conv): Conv1d(200, 200, kernel_size=(40,), stride=(1,), padding=(39,))
      (dropout): Dropout(p=0, inplace=False)
      (downsample): Linear(in_features=200, out_features=200, bias=True)
      (bn): BatchNorm1d(200, eps=1e-05, momentum=0.1, affine=True,
          track_running_stats=True)
    )
    (3): ConvBlock(
      (conv): Conv1d(200, 200, kernel_size=(40,), stride=(1,), padding=(39,))
      (dropout): Dropout(p=0, inplace=False)
      (downsample): Linear(in_features=200, out_features=200, bias=True)
      (bn): BatchNorm1d(200, eps=1e-05, momentum=0.1, affine=True,
          track_running_stats=True)
    )
    (4): ConvBlock(
      (conv): Conv1d(200, 200, kernel_size=(40,), stride=(1,), padding=(39,))
      (dropout): Dropout(p=0, inplace=False)
      (downsample): Linear(in_features=200, out_features=200, bias=True)
      (bn): BatchNorm1d(200, eps=1e-05, momentum=0.1, affine=True,
          track_running_stats=True)
    )
    (5): ConvBlock(
      (conv): Conv1d(200, 200, kernel_size=(40,), stride=(1,), padding=(39,))
      (dropout): Dropout(p=0, inplace=False)
      (downsample): Linear(in_features=200, out_features=200, bias=True)
      (bn): BatchNorm1d(200, eps=1e-05, momentum=0.1, affine=True,
          track_running_stats=True)
    )
  )
  (fc1): Linear(in_features=200, out_features=10, bias=True)
  (fc2): Linear(in_features=5030, out_features=100, bias=True)
  (g): Sequential(
    (0): Linear(in_features=100, out_features=25, bias=False)
    (1): BatchNorm1d(25, eps=1e-05, momentum=0.1, affine=True, track_running_stats=True)
    (2): ReLU(inplace=True)
    (3): Linear(in_features=25, out_features=128, bias=True)
  )
)
```

Fig. 7: Spiro-CLF model architecture. The model consists of six convolution blocks, each containing a 1D convolution layer with weight normalization, ReLU activation, and skip-connection. The convolution layers have a fixed kernel size of 40 with padding to avoid downsampling. After the convolution blocks, two fully-connected layers downsample to the 100-dimensional feature space. The model is trained using a contrastive loss function.

D.5 Evaluating Spiro-CLF Learned Representations

After training the Spiro-CLF model on the UKB training partition, we applied Spiro-CLF to the training and test partitions of the UKB and COPDGene datasets to generate the learned Spiro-CLF lung function representations. Note that the trained Spiro-CLF model is applied directly to the COPDGene dataset without additional finetuning on the COPDGene samples. We applied an additional Principal Component Analysis (PCA) procedure on the COPDGene representations and saved the first 24 principal components (99.1% of total variability), which improved results on COPDGene evaluation.

UKB and COPDGene representations were evaluated in a variety of tasks to assess the representation quality. We evaluated 1) binary prediction of lung function impairment (UKB, Sec. D.5.1), 2) Cox regression for all-cause mortality (UKB and COPDGene, Sec. D.5.2), and 3) Phenotype prediction (COPDGene, Sec. D.5.3).

In all experiments we used a bootstrap resampling procedure to estimate the standard error of the reported prediction performance. Specifically, we resampled ($n = 50$) the test partitions of each dataset to generate sampling distribution of each test metric. The 95% confidence intervals for the test metrics

are reported for each experiment. We used the Holm-Bonferroni method [37] to correct for multiple comparisons in significance testing for the downstream tasks.

D.5.1 Lung Function Impairment Prediction

We defined lung function impairment using two metrics: FEV_1/FVC and FEV_1 percent of predicted (FEV_1pp). We calculated FEV_1pp using GLI-2012 reference values [14] calculated using the Spiref[3] python package. We took sex, height, age at recruitment, and self-reported ethnicity values from fields 31, 12144, 21022, 21000. Self-reported ethnicities were mapped to “Caucasian” or “Other” to maintain consistency with GLI-2012 classifications.

To predict lung function impairment we created binary labels for each individual based on a threshold for each metric. Specifically we defined $FEV_1/FVC < 0.7$ and $FEV_1pp < 80\%$, the Global Initiative for Chronic Obstructive Lung Disease (GOLD) spirometric criteria for moderate-to-severe airflow limitation in COPD [38] as indicative of lung function impairment. Using these definitions we trained logistic regression models for each task using the Spiro-CLF features as input. The regression models were trained with no regularization and no additional covariates. Model performance was assessed using the Area under the Receiving Operating Characteristic (AUROC) curve on the test set.

D.5.2 Mortality Prediction

We used UK Biobank field 3060 to identify the date the recorded spirometry was performed and field 40000 for event time for all-cause mortality. The dataset contained 12,390 mortality events (3.5%) with a median time-to-event of 11 years.

We trained a Cox Proportional Hazards Regression model on the Spiro-CLF features extracted from the training set and evaluated the concordance index (c-index) on the Spiro-CLF test features. c-index is a goodness-of-fit measurement that evaluates the concordance between the labels and predicted outcomes. A higher c-index indicates a better model fit. We omitted any covariates in order to evaluate the predictive power of the Spiro-CLF features. Interestingly, incorporating feature averaging on the generated Spiro-CLF features reduced predictive performance for the Cox model. We therefore omitted this step in this predictive task (see Sec. 4 for additional discussion).

We compared the results from using the Spiro-CLF representation with previously proposed spirometry metrics, such as FEV_1, FVC, and the forced mid-expiratory flow between 25% and 75% of the FVC (FEF25-75). These alternative metrics were calculated on the maximal QC-passing effort for each individual. The set of metrics from the training dataset were then used as predictors in the Cox Regression model.

D.5.3 Phenotype Prediction

We used the learned Spiro-CLF represenions to predict 13 COPDGene phenotypes (Table 4) related to lung function. We combined the Spiro-CLF representations with three sets of predictors and measured the improvement in model fit due to Spiro-CLF:

1. **Intercept-only.** No independent variables were included.
2. **Spirogram Metrics.** We included traditional metrics FEV_1, FVC, and FEV_1/FVC, taken from each participant’s maximal QC-passing effort.
3. **Spirogram Metrics & Covariates.** In addition to the spirogram metrics, we included relevant covariates for each phenotype, as listed in Table 4. Covariates were selected based on prior work on phenotype association in COPDGene [15].

[3] https://github.com/kennethverstraete/spiref

Phenotype Name	Link Function	Metric	Covariates							
			Age	Gender	Race	PackYears	Height	SmokingStatus	FEV1pp	CT Scanner
Asthma	Logit	AUROC	x	x	x	x				
Chronic Bronchitis	Logit	AUROC	x	x	x	x			x	
Distance walked	Identity	MSE	x	x	x	x			x	
Inspiration $\leq$ 950	Identity	MSE	x	x	x	x		x	x	x
Lung density	Identity	MSE	x	x	x	x		x	x	
Pi10	Identity	MSE	x	x	x	x	x		x	x
PRM airtrapping%	Identity	MSE	x	x	x	x		x	x	x
PRM emphysema%	Identity	MSE	x	x	x	x		x	x	x
PRM normal%	Identity	MSE	x	x	x	x		x	x	x
SGRQ score	Identity	MSE	x	x	x	x			x	
Wall Area%	Identity	MSE	x	x	x	x	x		x	x
MMRC Dyspnea	Identity	MSE	x	x	x	x			x	
Exacerbation freq	Log	MSE	x	x	x	x		x	x	

Table 4: List of phenotypes used in the phenotype prediction experiment (Sec. D.5.3) with the link function of each respective GLM, test metric for evaluating performance, and selected covariates.

We trained two Generalized Linear Models (GLM) for each phenotype and predictor set, one without Spiro-CLF features and one with Spiro-CLF features included (m = 78 models in total). The GLMs were trained on the COPDGene training partition, and then evaluated on the test partition. The GLM link functions and evaluation metrics were selected based on phenotype data type (Table 4).

We evaluated the Spiro-CLF features by calculated the change in evaluation metric (AUROC or MSE) between models excluding and including Spiro-CLF features for each phenotype and set of predictors (Figure 6). We also calculated the joint significance of the Spiro-CLF features for each phenotype and set of predictors. The F-test significance results are discussed in App. F.5.

For the UKB dataset, Asthma labels were determined based on ICD9 and ICD10 codes. Chronic Bronchitis labels were determined by ICD9 and ICD10 codes, as well as self-reported chronic cough (field 22502).

D.6 Feature Space Analysis

We use a dimension-reduction method, Uniform Manifold Approximation and Projection (UMAP) [39], to project the Spiro-CLF representations down to two dimensions for visualization purposes. We randomly sampled 2000 participants and calculated the Spiro-CLF feature embeddings for the entirety of their respective efforts, including flow-time and flow-volume transformations. We then applied UMAP to project the 100-dimensional embeddings to two dimensions.

We evaluated the level of invariance induced by the Spiro-CLF model by investigating the feature space. The goal of the Spiro-CLF model training is to create a representation of lung function that is invariant to the inherent noise in repeated exhalation effort. An invariant representation would have minimal variance even when the individual efforts from a participant are not identical. We evaluated the empirical performance by calculating the average Euclidean distance in the feature space for each individual's efforts. We first calculated the average Spiro-CLF representation for each individual, then averaged the Euclidean distance between each individual representation and the average representation.

D.7 Feature Importance Approximation

Machine learning explainability methods have been increasingly relied upon to better understand complex deep learning models [40–42]. In particular, feature importance methods have been used to understand the relative importance of features or covariates for a given prediction, especially in the context of medical data [43–46].

Therefore, we applied feature importance methods to understand how Spiro-CLF and subsequent downstream prediction tasks operate on the spirogram samples. We first combined the Spiro-CLF model and downstream linear model for Lung Function impairment (Section D.5.1) into a single prediction pipeline. We then applied the Asymmetric Shapley Value (ASV) [47] method to calculate the feature importance values.

ASV has a number of properties that are suited for this application:

- *Post-hoc.* ASV can be applied to any prediction model after the model has been trained with no modification to the training process or model architecture.
- *Local.* ASV can be calculated for individual data samples. Traditionally, most feature importance methods are *global*, where they give a single explanation for the entire dataset and model. Using a local method allows the investigation of different subgroups within the data distribution by averaging over the relevant samples. For example, we can calculate ASV separately for flow-time, flow-volume, and volume-time transformations.
- *Asymmetric.* Due to the time-dependency in the spirometry efforts, the individual features within each sample contains asymmetric interactions. For example, in our dataset each feature represents a 50ms timestamp. Within each pair of timestamps, one timestamp occurs before the other. Therefore the relative importance of the latter timestamp is conditional on the former timestamp. We can enforce this relationship using ASV using a directed graph which we define prior to use.

We approximated ASV using a Monte Carlo sampling algorithm [48] with 2000 samples, implemented using the code provided in Masoomi et al. [49]. We applied this approximation on 50 randomly sampled spirometry efforts for each of flow-time, flow-volume, and volume-time transformations, then averaged over each transformation to obtain the subgroup-level explanation.

E Background

E.1 Contrastive Learning

Contrastive learning (CL) is an unsupervised method machine learning method to learn latent representations of a data distribution. CL methods have been popularized in domains such as computer vision, natural language processing, and time-series analysis [31]. In contrast to other unsupervised frameworks, such as Variational Autoencoders [24], or Generative Adversarial models [50], CL models use a discriminative approach that attempts to group similar samples closer together, and dissimilar samples further apart. Specifically, groups of similar and dissimilar samples are defined by the user, and the CL model is trained to recover the group structure. Therefore, there are three primary components when building a CL model: 1) Sample Groups, 2) Deep Learning Architecture, and 3) Loss Function.

1. Sample Groups. CL models rely on a dataset with predefined groups of similar samples. In many applications, ”similar” groups are synthetically generated from individual dataset samples through data transformations, also known as augmentations. For example, in computer vision, an image from the dataset can be transformed using random crops, rotations, or color adjustments. These transformations create multiple views of the same image, which are then treated as similar samples. This group of synthetically generated images, derived from the original sample, forms a set of “positive pairs” in the CL framework. Negative pairs, on the other hand, consist of unrelated images. These augmented samples serve as inputs to the CL model, helping it learn representations that capture the inherent characteristics of each data sample.

2. Deep Learning Architecture. Various deep learning architectures can be adapted for use in contrastive learning, each suited to different types of data and tasks. For example, Convolutional Neural Networks (CNNs) are commonly used for image-based contrastive learning tasks, as they can capture spatial features in visual data. CNN-based CL models learn image representations by contrasting augmented views of the same image with those of different images, thus forming meaningful visual embeddings. For

sequential data, such as text or time-series, Recurrent Neural Networks (RNNs) or CNNs are frequently employed. These architectures can capture temporal dependencies and are often used in contrastive learning tasks involving natural language processing or time-series forecasting.

3. Loss Function. Finally, the user must define a contrastive loss function calculates similarity between samples. One widely used formulation of contrastive loss is the Normalized Temperature-scaled Cross Entropy Loss (NT-Xent) [9], which has been popularized by frameworks like SimCLR. Let sim() represent cosine similarity, and $\mathbb{1}$ represent the indicator function. Then the NT-Xent loss between two feature vectors z_i, and z_j can be written as follows:

$$\mathcal{L}(z_i, z_j) = -\log \frac{\exp\left[\text{sim}(z_i, z_j)/\tau\right]}{\sum_{k=1}^{2N} \mathbb{1}_{k \neq i} \exp\left[\text{sim}(z_i, z_j)/\tau\right]} \tag{1}$$

The NT-Xent loss ensures that positive pairs, typically augmentations of the same data sample, have higher similarity scores, while negative pairs, which may represent different samples or augmentations, have lower similarity scores. This simple yet effective loss function drives the model to produce embeddings that capture essential characteristics of the data.

E.2 Cox Regression

Cox regression, or the Cox proportional hazards model, is a survival analysis method used to examine the relationship between time until an event, such as mortality, and one or more predictor variables. More concretely, the goal of the Cox model is to predict an individual's *hazard*, i.e. the probability of an event at time t, as a function of individual's covariates. Let t represent time, $X_1, \ldots, X_p$ represent covariates, and $\beta_1, \ldots, \beta_p$ represent linear coefficients. The Cox model assumes that the individual's hazard $h(t|X)$ is equal to the product of a baseline hazard $h_0(t)$ and a linear combination of the covariates.

$$h(t|X) = h_0(t) \exp[\beta_1 X_1 + \ldots + \beta_p X_p] \tag{2}$$

The Cox model does not assume any distribution of survival times. Instead, it assumes that the hazard ratios between individuals are constant over time and depend solely on the covariate values. The β coefficients in the Cox model are solved through maximum likelihood, and represent the the relative risk associated with each corresponding covariate.

E.3 Concordance Index

The concordance index (c-index), also known as the concordance statistic, is a metric commonly used to evaluate performance for survival models, especially Cox proportional hazards models, as well as other models in settings where the goal is to predict the order or rank of outcomes. Intuitively, the concordance index measures how well a model's predictions agree with the actual observed outcomes. More concretely, it is a measure of rank correlation between predicted risks scores and observed events. Higher values indicate better model performance. Similar to AUROC, a score of 1.0 representing perfect performance, while a score of 0.5 indicates random prediction.

F Supplemental Results

F.1 Understanding the Spiro-CLF Feature Space

In order to visualize the learned Spiro-CLF representations, we plotted a low-dimensional projection of the UKB feature samples. Given that the generated features are high-dimensional (100 dimensional vectors), we applied the Uniform Manifold Approximation and Projection (UMAP) [39] algorithm on the output of the Spiro-CLF model. The resulting feature space visualization is shown in Figure 8.

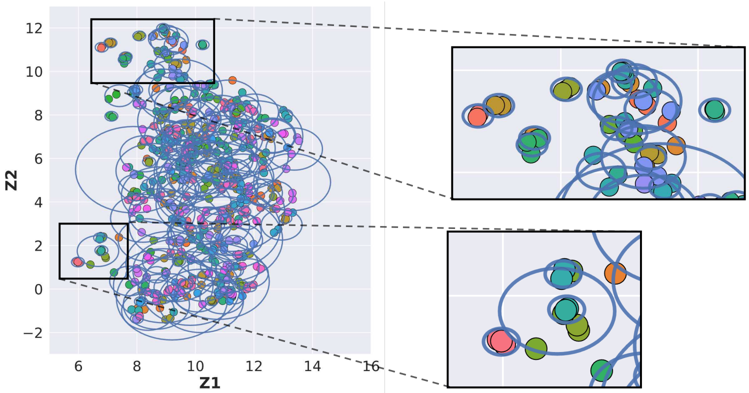

Fig. 8: Analysis of the Spiro-CLF feature space which shows that efforts from the same individual are mapped to be close together in the feature space. We projected the Spiro-CLF representations of a random subset of 500 individuals to two dimensions using the UMAP algorithm. Each individual's spirometry efforts were coded to be a different color and circled in blue.

Transformation	Volume-Time	Flow-Time	Flow-Volume
Volume-Time	0.128	0.282	0.215
Flow-Time	0.282	0.152	0.291
Flow-Volume	0.215	0.291	0.176

Table 5: Effects of different transformations on the corresponding feature space. The table shows average distance between the feature representations of each individual's efforts.

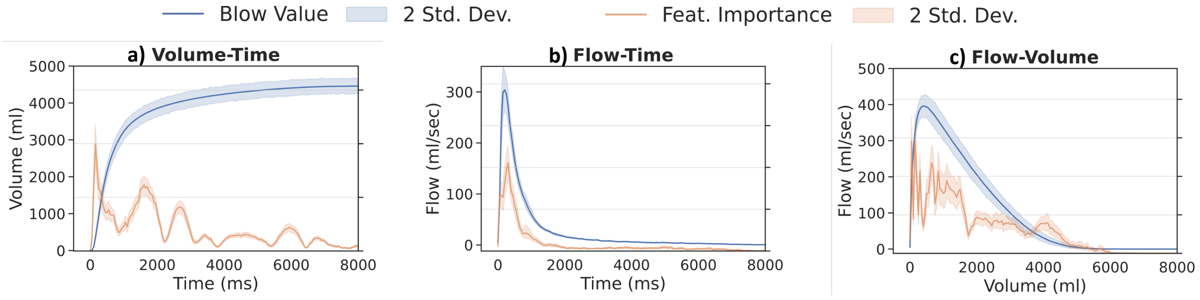

Fig. 9: Visualization of the relative importance of different sections of the spirometry curve with respect to the prediction of lung function impairment.

The Spiro-CLF model was trained using a contrastive loss, which pushes spirometry efforts from the same individual closer together in the feature space. In the low dimensional feature space, Spiro-CLF spirometry representations resulting from different efforts or different data transformation appeared to project to a small locality. This observation indicates that the Spiro-CLF model generated similar feature embeddings for different spirometry efforts (or different transformations) performed by the same individual. We evaluated this observation by measuring the average Euclidean distance in the feature space generated using our trained model. Figure 8 is a visualization of the clusters of each individual effort in the feature space. Each blue circle in the figure surrounds the features for a single individual. Table 5 calculates the distance between projections given different pairwise combinations of transformations. We observed that increasing the number of transformations increased the distance between clusters of feature projections. This indicates that adding transformations is a more difficult task for the Spiro-CLF model. Between the three investigated transformations, we observed that the flow-volume transformation yielded the largest average distance, which indicates that the model had the most difficulty in minimizing contrastive loss under this transformation.

F.2 Assigning Relative Importance to Spirometry Curves

We further applied machine learning interpretability methods to investigate how the Spiro-CLF deep learning model processes spirograms in downstream predictions. We applied the Asymmetric Shapley Value (ASV) [47] method to 50 randomized maximal spirograms in order to assign relative importance values to different segments of input spirograms (Fig. 9). The ASV results were averaged over sampled spirograms for each transformation. Importance measures are relative to the prediction of COPD in the downstream task. We observed that feature importance peaked within the first 1000 ms of the spirometry efforts, which is associated with peak expiratory flow (PEF) and the FEV_1. This section of the importance curve corresponds to low variance in importance estimate over the averaged samples. In the flow-volume figure (Fig. 9(c)) we observed a decrease in importance between 2000mL and 4000mL segment with another local peak at 4100mL. This section corresponds to higher variance. This section generally corresponds to FVC, with higher variance due to sampled efforts achieving FVC at different timestamps.

F.3 Comparison of FEV_1/FVC and FEV_1PP prediction using the second-highest effort

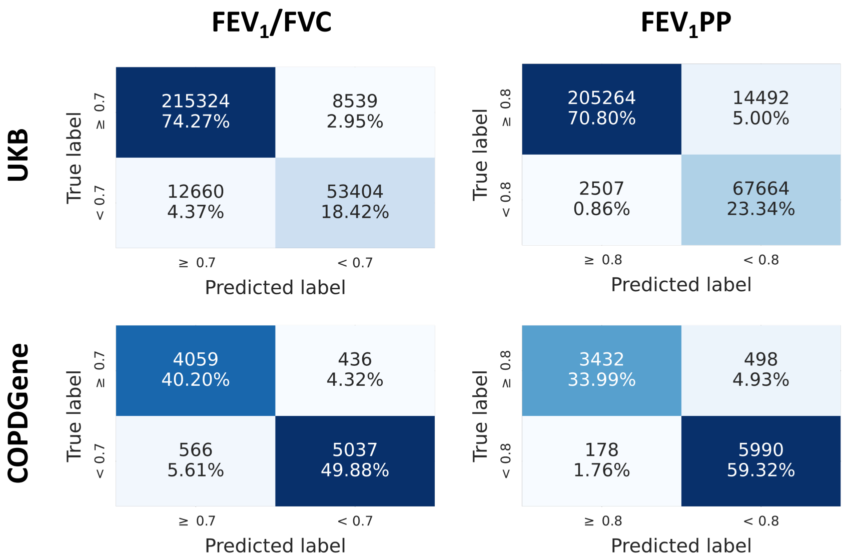

Fig. 10: Confusion matrix when using the FEV_1, FVC, and FEV_1PP of the second-highest, QC-passing effort to predict $FEV_1/FVC < 0.7$ and $FEV_1PP < 80\%$.

In Section 3.1 we tested the effectiveness of using the Spiro-CLF representation for the binary prediction of A) $FEV_1/FVC < 0.7$ and B) $FEV_1PP < 80\%$. However, the majority of QC-passing, submaximal efforts are within 250mL FVC of the maximal effort (Fig. 2). In Figure 10 we evaluated the effectiveness of using the second-highest effort (measured by FVC), in place of the maximal effort for the two binary prediction tasks. Specifically, we defined the ground-truth FEV_1/FVC and FEV_1PP values on the maximal effort and compared the agreement with the same values calculated on each individual's second-highest, QC-passing, effort. The results are shown in a confusion matrix. We observe that using the second-highest effort in UKB leads to misclassification rates of 7.3% (0.834 F1) for task A and 5.9% (0.888 F1) for task B. For COPDGene, we observe misclassification rates of 9.9% (0.910 F1) for task A and 6.7% (0.947 F1) for task B.

F.4 Comparison of $FEV_1/FVC < 0.7$ prediction using QC-failing and submaximal efforts in UKB

In this experiment, we compared the results of the Spiro-CLF prediction of $FEV_1/FVC< 0.7$ with the FEV_1/FVCvalues of each subset of efforts. Specifically, we calculated the FEV_1/FVC for individual efforts in each QC-failing and submaximal effort subset and used these values as predictions for the $FEV_1/FVC< 0.7$ prediction task. We evaluate results using the accuracy metric in place of AUROC due to the non-probabilistic property of the predictions.

F.5 COPDGene Phenotype Prediction: F-Test

We additionally evaluated each GLM in the phenotype prediction task (Sec. D.5.3) using an F-test to determine the combined statistical significance of the Spiro-CLF representation features. The results are shown in Table 6. The F-test results indicate that the Spiro-CLF representation features are jointly significant in predicting COPDGene phenotypes even when including the phenotype covariates and FEV_1, FVC, FEV_1/FVC metrics. This result implies that the Spiro-CLF representation is predictive of lung-related phenotypes, even after adjusting for covariates and traditional spirometry metrics.

F.6 Spiro-CLF Generalization over Held-Out Assessment Centers

The UKB dataset consists of participants from 21 assessment centers across the UK (Figure 12). In this experiment, we validate Spiro-CLF generalization results by stratifying over different assessment centers.

We first partition the UKB dataset, separating the 2 largest assessment centers, Bristol and Leeds, as a held-out test set. We combine the remaining assessment centers and split this into a training (80%) and validation (20%) set. We then retrain the Spiro-CLF model, using the same method described in Section 2. We apply the resulting trained model on the mortality and lung function impairment prediction tasks. Results are shown in Figure 13. We observe that the results follow the previously seen results when using the randomly sampled test partition.

G COPDGene Funding and Acknowledgements

COPDGene Phase 3

Grant Support and Disclaimer

The project described was supported by Award Number U01 HL089897 and Award Number U01 HL089856 from the National Heart, Lung, and Blood Institute. The content is solely the responsibility of the authors and does not necessarily represent the official views of the National Heart, Lung, and Blood Institute or the National Institutes of Health.

COPD Foundation Funding

COPDGene is also supported by the COPD Foundation through contributions made to an Industry Advisory Board that has included AstraZeneca, Bayer Pharmaceuticals, Boehringer-Ingelheim, Genentech, GlaxoSmithKline, Novartis, Pfizer, and Sunovion.

COPDGene® Investigators – Core Units

Administrative Center: James D. Crapo, MD (PI); Edwin K. Silverman, MD, PhD (PI); Barry J. Make, MD; Elizabeth A. Regan, MD, PhD

Genetic Analysis Center: Terri H. Beaty, PhD; Peter J. Castaldi, MD, MSc; Michael H. Cho, MD, MPH; Dawn L. DeMeo, MD, MPH; Adel El Boueiz, MD, MMSc; Marilyn G. Foreman, MD, MS; Auyon Ghosh, MD; Lystra P. Hayden, MD, MMSc; Craig P. Hersh, MD, MPH; Jacqueline Hetmanski, MS; Brian D. Hobbs, MD, MMSc; John E. Hokanson, MPH, PhD; Wonji Kim, PhD; Nan Laird, PhD; Christoph Lange, PhD; Sharon M. Lutz, PhD; Merry-Lynn McDonald, PhD; Dmitry Prokopenko, PhD; Matthew Moll, MD, MPH; Jarrett Morrow, PhD; Dandi Qiao, PhD; Elizabeth A. Regan, MD, PhD; Aabida Saferali, PhD; Phuwanat Sakornsakolpat, MD; Edwin K. Silverman, MD, PhD; Emily S. Wan, MD; Jeong Yun, MD, MPH

Imaging Center: Juan Pablo Centeno; Jean-Paul Charbonnier, PhD; Harvey O. Coxson, PhD; Craig J. Galban, PhD; MeiLan K. Han, MD, MS; Eric A. Hoffman, Stephen Humphries, PhD; Francine L. Jacobson, MD, MPH; Philip F. Judy, PhD; Ella A. Kazerooni, MD; Alex Kluiber; David A. Lynch, MB; Pietro Nardelli, PhD; John D. Newell, Jr., MD; Aleena Notary; Andrea Oh, MD; Elizabeth A. Regan, MD, PhD; James C. Ross, PhD; Raul San Jose Estepar, PhD; Joyce Schroeder, MD; Jered Sieren; Berend C. Stoel, PhD; Juerg Tschirren, PhD; Edwin Van Beek, MD, PhD; Bram van Ginneken, PhD; Eva van Rikxoort, PhD; Gonzalo Vegas Sanchez-Ferrero, PhD; Lucas Veitel; George R. Washko, MD; Carla G. Wilson, MS;

PFT QA Center, Salt Lake City, UT: Robert Jensen, PhD

Data Coordinating Center and Biostatistics, *National Jewish Health, Denver, CO*: Douglas Everett, PhD; Jim Crooks, PhD; Katherine Pratte, PhD; Matt Strand, PhD; Carla G. Wilson, MS

Epidemiology Core, *University of Colorado Anschutz Medical Campus, Aurora, CO*: John E. Hokanson, MPH, PhD; Erin Austin, PhD; Gregory Kinney, MPH, PhD; Sharon M. Lutz, PhD; Kendra A. Young, PhD

Mortality Adjudication Core: Surya P. Bhatt, MD; Jessica Bon, MD; Alejandro A. Diaz, MD, MPH; MeiLan K. Han, MD, MS; Barry Make, MD; Susan Murray, ScD; Elizabeth Regan, MD; Xavier Soler, MD; Carla G. Wilson, MS

Biomarker Core: Russell P. Bowler, MD, PhD; Katerina Kechris, PhD; Farnoush Banaei-Kashani, PhD

COPDGene® Investigators – Clinical Centers

Ann Arbor VA: Jeffrey L. Curtis, MD; Perry G. Pernicano, MD

Baylor College of Medicine, Houston, TX: Nicola Hanania, MD, MS; Mustafa Atik, MD; Aladin Boriek, PhD; Kalpatha Guntupalli, MD; Elizabeth Guy, MD; Amit Parulekar, MD;

Brigham and Women's Hospital, Boston, MA: Dawn L. DeMeo, MD, MPH; Craig Hersh, MD, MPH; Francine L. Jacobson, MD, MPH; George Washko, MD

Columbia University, New York, NY: R. Graham Barr, MD, DrPH; John Austin, MD; Belinda D'Souza, MD; Byron Thomashow, MD

Duke University Medical Center, Durham, NC: Neil MacIntyre, Jr., MD; H. Page McAdams, MD; Lacey Washington, MD

HealthPartners Research Institute, Minneapolis, MN: Charlene McEvoy, MD, MPH; Joseph Tashjian, MD

Johns Hopkins University, Baltimore, MD: Robert Wise, MD; Robert Brown, MD; Nadia N. Hansel, MD, MPH; Karen Horton, MD; Allison Lambert, MD, MHS; Nirupama Putcha, MD, MHS

Lundquist Institute for Biomedical Innovation at Harbor UCLA Medical Center, Torrance, CA: Richard Casaburi, PhD, MD; Alessandra Adami, PhD; Matthew Budoff, MD; Hans Fischer, MD; Janos Porszasz, MD, PhD; Harry Rossiter, PhD; William Stringer, MD

Michael E. DeBakey VAMC, Houston, *TX*: Amir Sharafkhaneh, MD, PhD; Charlie Lan, DO

Minneapolis VA: Christine Wendt, MD; Brian Bell, MD; Ken M. Kunisaki, MD, MS

Morehouse School of Medicine, Atlanta, GA: Eric L. Flenaugh, MD; Hirut Gebrekristos, PhD; Mario Ponce, MD; Silanath Terpenning, MD; Gloria Westney, MD, MS

National Jewish Health, Denver, CO: Russell Bowler, MD, PhD; David A. Lynch, MB

Reliant Medical Group, Worcester, MA: Richard Rosiello, MD; David Pace, MD

Temple University, Philadelphia, PA: Gerard Criner, MD; David Ciccolella, MD; Francis Cordova, MD; Chandra Dass, MD; Gilbert D'Alonzo, DO; Parag Desai, MD; Michael Jacobs, PharmD; Steven Kelsen, MD, PhD; Victor Kim, MD; A. James Mamary, MD; Nathaniel

Marchetti, DO; Aditi Satti, MD; Kartik Shenoy, MD; Robert M. Steiner, MD; Alex Swift, MD; Irene Swift, MD; Maria Elena Vega-Sanchez, MD

University of Alabama, Birmingham, AL: Mark Dransfield, MD; William Bailey, MD; Surya P. Bhatt, MD; Anand Iyer, MD; Hrudaya Nath, MD; J. Michael Wells, MD

University of California, San Diego, CA: Douglas Conrad, MD; Xavier Soler, MD, PhD; Andrew Yen, MD

University of Iowa, Iowa City, IA: Alejandro P. Comellas, MD; Karin F. Hoth, PhD; John Newell, Jr., MD; Brad Thompson, MD

University of Michigan, Ann Arbor, MI: MeiLan K. Han, MD MS; Ella Kazerooni, MD MS; Wassim Labaki, MD MS; Craig Galban, PhD; Dharshan Vummidi, MD

University of Minnesota, Minneapolis, MN: Joanne Billings, MD; Abbie Begnaud, MD; Tadashi Allen, MD

University of Pittsburgh, Pittsburgh, PA: Frank Sciurba, MD; Jessica Bon, MD; Divay Chandra, MD, MSc; Joel Weissfeld, MD, MPH

University of Texas Health, San Antonio, San Antonio, TX: Antonio Anzueto, MD; Sandra Adams, MD; Diego Maselli-Caceres, MD; Mario E. Ruiz, MD; Harjinder Singh

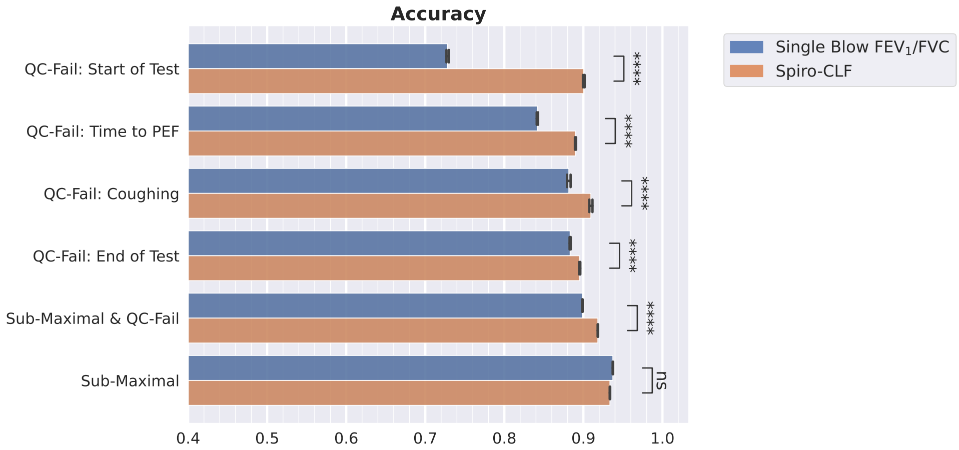

Fig. 11: Accuracy for $FEV_1/FVC < 0.7$ prediction task using QC-failing and submaximal efforts in UKB. We compared the performance of the Spiro-CLF representation and the raw single-effort FEV_1/FVC of each subset of efforts. The error bars represent 95% bootstrap confidence intervals (50 iterations). **** indicates significance at the $\alpha = 10^{-4}$ significance level, with Holm-Bonferroni adjustment.

Phenotype Name	Link Function	Base Predictors			
		Intercept-Only	Covariates	Spirogram Metrics	Covariates & Spirogram Metrics
**UKB**					
Asthma	Logit	***	***	***	***
Chronic Bronchitis	Logit	***	***	***	***
**COPDGene**					
Asthma	Logit	***	***	***	***
Chronic Bronchitis	Logit	***	***	***	***
Distance walked	Identity	***	***	***	***
Inspiration $\leq$ 950	Identity	***	***	***	***
Lung density	Identity	***	***	***	***
Pi10	Identity	***	***	***	***
PRM airtrapping%	Identity	***	***	***	***
PRM emphysema%	Identity	***	***	***	***
PRM normal%	Identity	***	***	***	***
SGRQ score	Identity	***	***	***	***
Wall Area%	Identity	***	***	***	***
MMRC Dyspnea	Identity	***	***	***	***
Exacerbation freq	Log	***	***	***	***

Table 6: F-Test Significance Results. *** represents signficance at the $\alpha = 10^{-3}$ significance level, with Holm-Bonferroni adjustment.

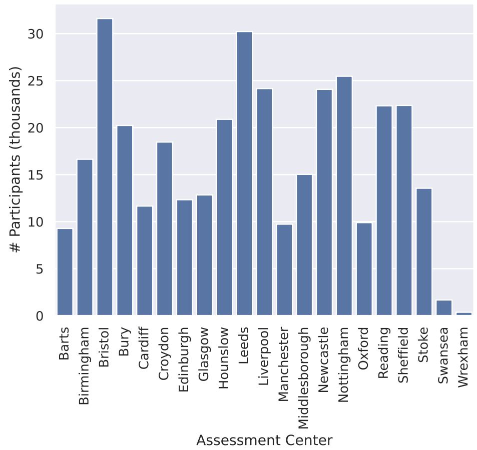

Fig. 12: Number of participants by assessment center (in thousands).

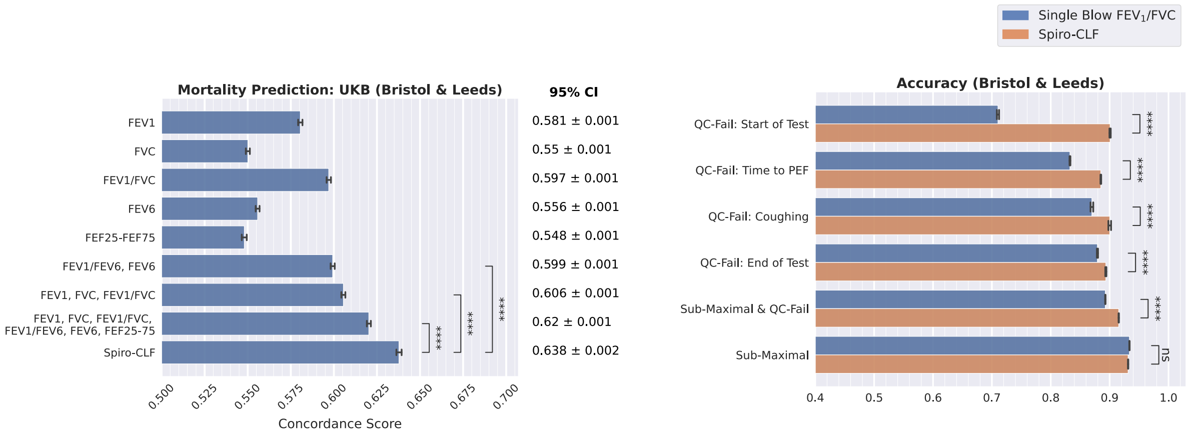

Fig. 13: Spiro-CLF results on the held-out Bristol & Leeds assessment centers.
